# Midlife heart rate variability and cognitive decline: A large longitudinal cohort study

**DOI:** 10.1016/j.ijchp.2024.100518

**Published:** 2024-11-20

**Authors:** Vera K. Jandackova, Shaun Scholes, Annie Britton, Andrew Steptoe

**Affiliations:** a Department of Epidemiology and Public Health, Faculty of Medicine, University of Ostrava, Syllabova 19, 703 00 Ostrava, Czech Republic; b Department of Human Movement Studies, Faculty of Education, University of Ostrava, City Campus Cerna Louka; Moravska Ostrava 3397, 702 00 Ostrava, Czech Republic; c Department of Epidemiology and Public Health, University College London, 1-19 Torrington Place, London WC1E 7HB, United Kingdom; d Department of Behavioural Science and Health, University College London, 1-19 Torrington Place, London WC1E 7HB, United Kingdom

**Keywords:** Autonomic nervous system, Cognitive decline, Aetiological significance, Heart rate variability, Longitudinal cohort studies

## Abstract

**Background:**

Autonomic dysfunction is common in dementia, yet its contribution to neurocognitive changes remains unknown. We investigated whether midlife cardiac vagal modulation, indexed by heart rate variability, associates with subsequent cognitive decline in adults without prior coronary heart disease or stroke.

**Methods:**

The sample comprised 2702 (1924 men) individuals initially aged 44–69 years from the UK Whitehall II cohort. Data from the fifth (1997–1999), seventh (2002–2004) and ninth (2007–2009) phases were analysed. Global cognitive function was ascertained from tests assessing memory, reasoning, vocabulary, and fluency. We used 12-lead-ECG-based heart rate variability measures, that primarily reflect vagal modulation (i.e. RMSSD and HF-HRV). Linear mixed-effects models and logistic regression were employed.

**Results:**

Results showed consistent associations between both vagally-mediated HRV measures and faster decline in global cognitive function. Specifically, low RMSSD and HF-HRV (lowest versus upper four quintiles) were associated with 0.07 SD (95% CI: -0.13, -0.01) and 0.06 SD (95% CI: -0.12, -0.004) accelerated 10-year cognitive decline after sociodemographic adjustments and faster decline in older ages. Further adjustments for lifestyle factors, medication use and other cardiometabolic conditions did not change the findings. Cognitive decline in individuals with low RMSSD and HF-HRV was estimated to progress 3 and 3.5 years faster per decade, respectively, compared to their counterparts. Additionally, participants with low RMSSD had 37% higher odds of low cognitive function (lowest quintile) at follow-up (OR 1.37: 95% CI,1.03, 1.80).

**Conclusion:**

Our findings support the aetiological significance of the autonomic nervous system, specifically vagal modulation, in the processes of cognitive decline and neurodegeneration. Low heart rate variability emerges as a potential biomarker indicative of acclerated cognitive decline that may extend over decades.

## Introduction

An estimated 55 million people worldwide are affected by dementia, and this is expected to triple by 2050 due to population ageing (Gauthier, Rosa-Neto, Morais & Webster, 2021). Cognitive decline and impairment, recognised as prodromal stages of dementia, pose significant public health challenges (Wilson, Leurgans, Boyle & Bennett, 2011). While no cure exists, managing modifiable risk factors could prevent or delay up to 40% of cases (Livingston et al., 2020). Increasing evidence links cardiovascular diseases (CVD) to dementia and cognitive changes, possibly through common risk factors and biological pathways (Cortes-Canteli & Iadecola, 2020). Autonomic dysfunction, including decreased vagal modulation of parasympathetic activity and/or increased sympathetic activity, observed in individuals with dementia subtypes and cognitive problems, is among the proposed biological mechanisms (Hase et al., 2020).

Heart rate variability (HRV) measurement is a valid, noninvasive technique for estimating autonomic nervous system (ANS) characteristics (Laborde, Mosley & Thayer, 2017; Shaffer & Ginsberg, 2017; Task Force , 1996). The root mean square of successive differences of normal-to-normal R-R intervals (RMSSD) and high-frequency HRV (HF-HRV), which reflects respiratory sinus arrhythmia, are particularly important because they relate predominantly to cardiac vagal modulation. A recent microneurographic study confirmed that vagus nerve firing patterns in the cervix align with neurons that modify cardiac function, including those responsible for respiratory sinus arrhythmia, offering neural validation for HRV as a marker of vagal modulation of heart rate (Farmer et al., 2024). Reduced HRV and diminished vagal cardiac modulation have been identified as predictors of cardiac morbidity and higher mortality, and indicators of stress vulnerability and low capacity for parasympathetic inhibition of autonomic arousal (Dekker et al., 2000; Jarczok et al., 2022; La Rovere et al., 2003; Task Force, 1996). A positive cross-sectional association between cognition and HRV has been demonstrated in many (Forte, Favieri & Casagrande, 2019; Frewen et al., 2013) but not all (Britton et al., 2008) large studies of middle-aged-to-older adults, as well as in smaller samples of individuals affected by mild cognitive impairment (MCI) or dementia (Collins, Dillon, Finucane, Lawlor & Kenny, 2012). A recent systematic review and meta-analysis of 27 studies found significant moderate associations between higher HRV, including RMSSD and HF-HRV, and better cognitive and behavioural outcomes in neurodegenerative disorders (Liu, Elliott, Knowles & Howard, 2022). Similarly a recent systematic review and meta-analysis found that patients with dementia or neurocognitive disorders had lower HRV, reflecting reduced parasympathetic activity, than healthy controls (Cheng, Huang & Huang, 2022). There is also evidence that vagal withdrawal precedes clinical presentation of dementia; in the Framingham cohort (age≥60years), RMSSD, a marker of vagal modulation, was associated with dementia risk over 10 years follow-up (Weinstein, Davis-Plourde, Beiser & Seshadri, 2021). Nevertheless, dementia represents a late stage of neuropathologic processes that may have progressed for decades (Verlinden et al., 2016; Wilson et al., 2011), raising questions as to whether decreased autonomic modulation is a result of neurodegeneration or whether it contributes to neuronal deterioration and cognitive decline. Epidemiological studies suggest cognitive decline may start in midlife (Singh-Manoux et al., 2012), and is a warning sign of potential future MCI and dementia (Verlinden et al., 2016; Wilson et al., 2011). Tracking autonomic regulation and cognitive changes from midlife onwards could offer insights into the role of autonomic dysfunction in cognitive decline. Investigations of longitudinal associations between cognitive function and HRV measurement have been mainly limited by the following reasons: (1) the use of older age samples (age≥75years) in which preclinical dementia cannot be ruled out (Mahinrad et al., 2015); (2) relatively short periods of follow-up (4–5 years) that do not permit tracking of cognitive change over long periods (Britton et al., 2008; Zeki Al Hazzouri et al., 2018); (3) not meeting well established guidelines for the short-term measurement of HRV (Schaich et al., 2020); and (4) lack of baseline cognitive performance data that prevents investigations of the association between HRV and the rate of cognitive change over time (Schaich et al., 2020).

To uncover the role of autonomic function in neurocognitive health, we used sensitive cognitive measures collected at three time points over 10 years in a large sample of middle-aged and older adults (the Whitehall II cohort). Our main goal was to investigate whether vagal withdrawal in midlife, indexed by low HRV, associated with subsequent rate of cognitive decline. Additionally we explored whether low HRV at midlife was associated with higher odds of low cognitive performance at follow-up.

Given the established link between CVD and cognitive impairment, potentially mediated by reduced cerebral blood flow and impaired brain vascular homeostasis (Cortes-Canteli & Iadecola, 2020; Yaffe et al., 2014), we considered CVD as a potential confounding factor. To mitigate this, we focused our analysis on participants without baseline coronary heart disease (CHD) and stroke diagnoses. This approach helps elucidate the potential empirical associations between HRV and cognitive decline, independent of history of any CVD.

## Methods

### Study population

**Fig. 1:**
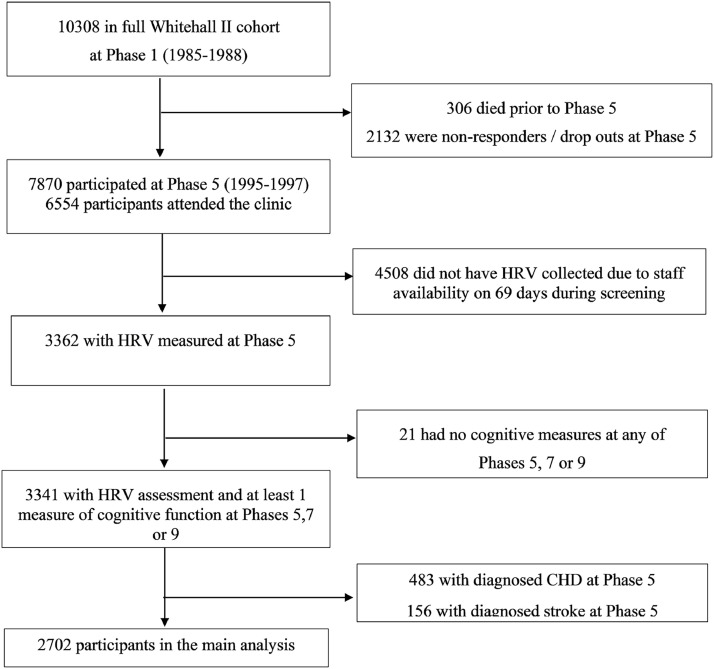
Study flowchart for the main analysis.

Whitehall II is a cohort study of men and women initially employed by the British civil service (Marmot & Brunner, 2005). The target population was all London-based office staff, aged 35–55 years. Fig. 1 shows the study flowchart. A total of 10,308 individuals (3413 women, response rate of 73%), were initially recruited between 1985 and 1988. At phase 5, all study members known to be alive and resident in the UK were invited to attend a screening clinic. Although 6554 participants attended the clinic at phase 5, HRV was recorded only for 3362 participants because of technical staff availability. No HRV recordings were collected on 69 days during screening, accounting for the majority of missing HRV data at phase 5. Participants who did not undergo HRV recordings at phase 5 did not differ significantly from those who did with respect to age, sex and employment grade (Hemingway et al., 2005). The University College London ethics committee approved the Whitehall II study and participants gave informed consent. Whitehall II data, protocols and other metadata are available to bona fide researchers for research purposes (details on the data sharing policy are available at

https://www.ucl.ac.uk/psychiatry/research/mental-health-older-people/whitehall-ii/data-sharing).

For the purposes of the present study we used cognitive test battery data administered at three clinical examinations over 10 years (phase 5:1997–1999, phase 7: 2002–2004, phase 9: 2007–2009) and HRV data administered at the fifth phase (1997–1999) of data collection. We used data on participants with no reported history of diagnosed CHD or stroke up to phase 5. The sample for the main analysis consisted of 2702 participants with HRV data at baseline (phase 5) and cognitive data in at least one of the three data collection phases (phases 5, 7, and 9).

### Heart rate and heart rate variability

Full details of the assessments of HRV in Whitehall II can be found elsewhere (Britton et al., 2007). Briefly, five-minute supine resting 12-lead electrocardiograms (ECG) were obtained after five minutes of rest using a Kardiosis device (Kardiosis Cardiologic Diagnostic Systems) at phase 5. The tachograms, which describe the sequences of RR intervals (the time between two successive R waves of the QRS complex), were visually inspected and exported from Kardiosis Cardiologic Diagnostic Systems. Five-minutes of beat-to-beat heart rate (HR) data were sampled at 500 Hz frequency to obtain a digitised sequence of R waves. An automatic algorithm specifically developed for Whitehall II HRV data (Acar, Savelieva, Hemingway & Malik, 2000) was used to detect ECG abnormalities including ectopic beats, right bundle-branch block, respiratory arrhythmia, blocked atrial extrasystole, and high-amplitude and wide T waves. Normal sinus-rhythm QRS complexes suited for a reliable HRV analysis were identified. Although spectral HRV analysis is prefered for short-term recordings obtained under standardised conditions (Task Force, 1996), both the time- and frequency-domain HRV analyses were conducted in this study. A time-domain measure was a root mean square of successive differences of normal-to-normal RR intervals (RMSSD; ms). Frequency-domain components were computed using the autoregressive method (the Blackman–Tukey algorithm), and for this study high-frequency 0.15 to 0.4 Hz (HF-HRV; ms^2^) spectral power was used. While RMSSD is highly correlated with HF-HRV, and both reflect vagal modulation of parasympathetic activity, the former may be less affected by alterations in respiration (Lahiri, Kannankeril & Goldberger, 2008; Penttilä et al., 2001; Task Force, 1996).

### Cognitive function

The cognitive test battery administered at baseline (1997–1999) and then at two 5-year intervals (2002–2004 and 2007–2009) over the next 10 years was chosen to provide a comprehensive assessment of cognitive function. The battery is appropriate for middle-aged adults and allows discrimination between low and high cognitive task performance (Singh-Manoux et al., 2012). The following cognitive function measures have been assessed (Sabia et al., 2012): verbal memory, verbal and mathematical reasoning, crystalised verbal intelligence and phonemic and semantic fluency. The tests included had high test–retest reliability (range 0.60–0.89) assessed on 556 Whitehall II participants who were invited back to the clinic within three months of having taken the test at phase 5 (Singh-Manoux et al., 2012).

Memory was measured by a short-term verbal memory test where participants were at 2-second intervals presented with a list of 20 one- or two-syllable words, randomly selected from a list of common English words (Marzano, Kendall & Paynter, 2005). Afterwards they were asked to write down immediately as many words as they could remember in any order that they wished (i.e. immediate free recall, a standard procedure for testing short-term memory) (Lezak, Howieson & Loring, 2004). Reasoning was assessed using The Alice Heim 4-I (AH4-I) test, composed of a series of 65 verbal and mathematical reasoning items of increasing difficulty (Heim, 1970). This test of inductive reasoning is a test of fluid general mental abilities measuring the ability to identify patterns and infer principles and rules. Participants had 10 min to complete this section. Crystallised verbal intelligence was measured using the Mill Hill vocabulary test. This test assesses knowledge of verbal meaning and encompasses the ability to recognise and comprehend words (Raven, 1965). The test was used in its multiple format, a list of 33 stimulus words ordered by increasing difficulty and six response choices. Finally, two measures of verbal fluency were used: phonemic fluency was assessed via ‘‘s’’ words and semantic fluency via ‘‘animal’’ words (Borkowski, Benton & Spreen, 1967). Participants were asked to recall in writing as many words beginning with ‘‘s’’ and as many animal names as they could. One minute was allowed for each test.

We standardised the raw scores for these five cognitive tests to z-scores (mean [SD]=0 [1]) to ease comparison between tests with different score ranges. As performance on tests of cognitive abilities are positively inter-related (Deary, 2012), we computed a single global cognitive function score by taking the average of the five standardised test scores. Composite scores of this sort are able to describe a much wider range of cognitive performance than individual tests, while minimising ceiling- and floor-effects and other forms of measurement error (Singh et al., 2000). Higher scores denoted better cognitive task performance. The Mini-Mental-State-Examination (MMSE) (Folstein, Folstein & McHugh, 1975), a brief 30-point measure of global cognition, was also used to differentiate ‘possible dementia’ or clinical cognitive impairment from non-clinical cognitive states. A conventional cut-point of <24 was used to classify participants as having mild cognitive impairment (Creavin et al., 2016). Because of ceiling effects in middle-aged populations (Folstein et al., 1975) and a limited number of individuals (*n* = 787) with baseline MMSE assessment (phase 5), the global cognitive score, rather than the MMSE score, was used as the dependent variable in our main analyses of change in global cognition.

### Confounders

We adjusted for relevant confounders (all assessed at phase 5) that have been previously shown to be associated with HRV or cognitive function (Britton et al., 2007; Jandackova, Scholes, Britton & Steptoe, 2016): age, sex, ethnicity (white vs. non-white), education (age left full-time education: less than 17 years; 17–18years; over 18 years), lifestyle factors, cardiovacular risk factors (other than diagnosed CHD or stroke) and prescribed medication use (Jandackova, Britton, Malik & Steptoe, 2016, 2019; Nolan, Jong, Barry-Bianchi, Tanaka & Floras, 2008). Lifestyle factors included leisure-time physical activity, alcohol consumption and smoking status, and were assessed by self-completion questionnaires. Participants were asked how often they took part in moderate- and vigorous-exercises such as walking, cycling, sports, gardening, housework and home maintenance. Those participants undertaking less than 1 h per week of vigorous physical activity or less than 2.5 h per week of moderate physical activity did not meet World Health Organization recommendations (Who Guidelines on Physical Activity & Sedentary Behaviour, 2020), and were defined as doing insufficient physical activity (Sabia et al., 2009). Alcohol consumption in the past week was expressed in units of alcohol (1 unit = 8 g), and ≥14 units/week was categorized as high consumption (in both sexes). Smoking status was categorised as non/ex/current smoker.

Cardiovascular risk factors included hypertension (blood pressure ≥160/90 mmHg), diagnosed diabetes, obesity (BMI ≥ 30kg/m^2^) and depressive symptoms (Ng, Turek & Hakim, 2013). Symptoms of depression were measured using a four-item scale derived from the 30-item General Health Questionnaire (GHQ; (Goldberg & Hillier, 1979)). Participants were classified as depressed if they scored ≥4 on the GHQ depression subscale or reported the use of prescribed antidepressant medication (Stansfeld, Head, Fuhrer, Wardle & Cattell, 2003). Lastly, medication use included any use of prescribed medication within the last 14 days.

### Statistical analyses

All analyses were conducted using Stata (SE 15.1). Participant characteristics were described as percentages or mean (SD or SE) when appropriate. Sociodemographic differences between the HRV groups (see below) were estimated using linear regression for continuous variables and Chi-Square tests for categorical variables. To examine the longitudinal association between baseline HRV and change in cognitive function between phase 5 and phase 9 (3 assessments, 1997–1999, 2002–2004, 2007–2009), we used data from 2702 participants with valid HRV data at baseline and with at least 1 assessment of cognitive performance over the follow-up period, and with no reported diagnosed stroke or CHD at baseline (phase 5). To examine the longitudinal association between baseline HRV and odds of low cognitive function at follow-up (phase 9), we used data from 2189 participants with valid HRV data at baseline and with assessment of cognitive performance at follow-up (phase 9, 2007–2009).

Given that there are no universally agreed criteria judging low autonomic function based on HRV, in order to assign its clinical significance, distributional cut-points may be a useful option. Therefore, we categorised HRV values into quintiles, an approach used previously in relation to cognition (Frewen et al., 2013). For ease of interpretation, for measures of HRV, we further classified participants into those in the lowest quintile (low HRV) versus the four upper quintiles (rest) based on sex- and age-specific values (44–54; 55–69 years). For HR, participants were classified as being in the highest age- and sex-specific quintile (high HR) versus the four lower quintiles (rest). The age- and sex-specific quintile values are listed in the footnote of Table 1.

**Table 1:** Descriptive characteristics of the sample at baseline (phase 5: 1997–1999) by the main heart rate variability measures (lowest vs upper 4 quintiles).

	RMSSD quintile, ms			HF-HRV quintile, ms^2^		
	Lowest (20%)	Upper 4 (80%)	*p*	Lowest (20%)	Upper 4 (80%)	*p*
**N**	541	2161		541	2161	
**Age (years), mean (SE)**	56.1 (0.3)	55.5 (0.1)	0.052	56.2 (0.3)	55.5 (0.1)	0.013
**Men, n (%)**	385 (71.7)	1539 (71.9)	0.981	385 (71.7)	1539 (71.9)	0.981
**White ethnic origin, n (%)**	508 (93.9)	1984 (91.8)	0.104	509 (94.1)	1983 (91.8)	0.071
**High educational level, n (%)**	136 (36.1)	643 (44.9)	0.002	140 (37.6)	639 (44.5)	0.018
**Lifestyle factors**						
Current smoker, n (%)	56 (10.5)	189 (8.9)	0.445	60 (11.2)	185 (8.7)	0.170
Insufficient physical activity, n (%)	413 (78.4)	1572 (74.6)	0.073	411 (78.1)	1574 (74.7)	0.099
High alcohol intake, n (%)	216 (41.2)	772 (36.8)	0.062	219 (42.1)	769 (36.6)	0.020
**Cardiovascular risk factors**						
Hypertension, n (%)	116 (21.4)	241 (11.2)	≤ 0.001	108 (20.0)	249 (11.5)	≤ 0.001
Diabetes, n (%)	18 (3.3)	39 (1.8)	0.028	18 (3.3)	39 (1.8)	0.028
Obesity (BMI > 30kg/m^2^), n (%)	114 (21.1)	221 (10.2)	≤ 0.001	111 (20.5)	224 (10.4)	≤ 0.001
Depressive symptoms, n (%)^a^	78(16.3)	281 (13.5)	0.718	79 (15.3)	275 (13.2)	0.200
**Prescribed medication use, n (%)**	224 (41.6)	787 (36.7)	0.036	229 (42.6)	782 (36.5)	0.009
Global cognitive score (z-standardized), mean (SE)	−0.03 (0.03)	0.04 (0.02)	0.037	−0.02 (0.03)	0.04 (0.02)	0.096
Memory, mean (SE)	6.8 (0.11)	6.9 (0.05)	0.353	6.8 (0.11)	6.9 (0.05)	0.246
Alice Heim Test -Reasoning, mean (SE)	46.3 (0.5)	46.6 (0.6)	0.502	46.1 (0.5)	46.7 (0.25)	0.326
Mill Hill Vocabulary, mean (SE)	24.7 (0.2)	25.0 (0.1)	0.142	24.8 (0.2)	25.0 (0.1)	0.449
Phonemic Fluency, mean (SE)	16.3 (0.2)	16.9 (0.1)	0.003	16.4 (0.2)	16.9 (0.1)	0.046
Semantic Fluency, mean (SE)	16.1 (0.2)	16.4 (0.1)	0.104	16.1 (0.2)	16.4 (0.1)	0.212

HF-HRV, high-frequency HRV; RMSSD, root mean square of successive differences of normal-to-normal RR intervals; SE., standard error;.

a GHQ score > 4 or reported use of antidepressants The cut-off for the lowest RMSSD quintile was <13.9 ms and <15 ms, respectively, in men and women aged 44 to 54 years and <10.5 ms and <11.6 ms, respectively, in men and women aged 55 to 69 years. The cut-off for the lowest HF-HRV quintile was <65.2 ms^2^ and <80.1 ms^2^, respectively, in men and women aged 44 to 54 years and <38.4 ms^2^ and <42.6 ms^2^, respectively, in men and women aged 55 to 69 years.

#### Main analysis: global cognitive function score

We adopted a linear mixed-effects models approach in our main analysis (Rabe-Hesketh & Skrondal, 2012) with cognitive function modelled as a continuous variable. Mixed-effects models use all available data over the follow-up, and take into account the fact that repeated measures on the same individual are positively correlated with each other, and can handle non-monotone missing data on the outcome. Random intercepts and slopes were included (via random effects) to account for unexplained variation between participants in baseline cognitive scores and in its rate of change, respectively; an additional term estimated intercept-by-slope covariance. The fixed slope for time-in-study in these models yields an estimate of the 10-year decline in the standardised cognitive score with associated 95% confidence intervals (95% CI).

The two HRV measures (RMSSD and HF-HRV) and heart rate were analysed as predictors in separate models of the global cognitive score. Each model included the following terms: (i) HRV (or heart rate) at phase 5 fitted as a time-invariant binary variable (HRV: lowest versus upper 4 quintiles; heart rate: highest versus lower 4 quintiles); (ii) time-in-study (exact time in years between phases, included as a continuous variable, divided by 10 to yield estimates of change in cognition over 10 years); (iii) the interaction term between the HRV categories (or heart rate) and time; (iv) age-at-baseline (treated as time-invariant); (v) the interaction term between age-at-baseline and time; (vi) the chosen sociodemographic variables: sex, ethnicity and educational level (all time-invariant) and (vii) their interaction with time. Men and women were combined in the analyses presented herein as the three-way interaction term between time-in-study, sex and HRV/heart rate categories, suggested no significant differences between men and women in the associations between HRV/heart rate and the 10-year rate of cognitive decline (all *p* between 0.378 and 0.653).

We explored the associations between HRV/heart rate and cognitive function (global test score) in a sequence of models. First, we adjusted for age, sex, ethnicity and education and their interaction with time (Model 1). For HRV, in Model 2, we additionally included heart rate and its interaction with time to examine the influence of heart rate on the estimated HRV and cognitive function associations. In further analyses, using Model 1 as the baseline, models additionally included lifestyle factors (Model 3), cardiovascular risk factors other than diagnosed CHD or stroke (hypertension, diabetes, obesity, and depression) (Model 4), prescribed medication use (Model 5), and finally all variables (Model 6). Each confounder was represented by a main effect and an interaction term with time-in-study, enabling differences in the baseline cognitive function score and in its rate of change. As a sensitivity analysis, we entered the HRV measures in the models as continuous rather than binary variables. Additional models were also fitted using the five separate cognitive function measures as outcome.

#### Additional analysis: low cognitive performance at follow-up

In additional analysis, assessing the robustness of the estimated associations between HRV and cognition, we tested whether HRV measurements at baseline were associated with higher odds of low cognitive performance at follow-up. Low cognitive function was defined as being in the lowest (sex- and age-specific) quintile of the global cognitive score. Logistic regression was used to model associations between HRV and the odds of low cognitive function at follow-up (phase 9, 2007–2009). Model 1 adjusted for sex, age, ethnicity and educational level; Model 2 additionally adjusted for health behaviours (smoking, alcohol, physical activity), obesity, depressive symptoms, and medication use at phase 5.

#### Sensitivity analysis

Two sensitivity analyses were conducted. First, to minimise the possibility of reverse causation, we repeated the analyses on a subset of healthy participants (*n* = 1779) who were free of MCI (MMSE >24) and diagnosed diabetes, obesity, hypertension and depressive symptoms. Secondly, to assess the robustness of the estimates of cognitive decline according to presence of potential autonomic dysfunction, we used different thresholds (25% and 33% ie, lowest quartile and lowest tertile) for autonomic dysfunction.

## Results

Fig. 1 shows the number of eligible participants included in the main analysis. Of the 10,308 participants at phase 1 (1985–1988), 306 had died and 2132 had dropped out from the study or did not respond before the start of the HRV and cognitive data collection at phase 5 (1997–1999). Of the remaining participants, 6554 participants (1909 women) attended the clinic (67% of participants), however HR was recorded for only 3365 participants due to staff availability (see the Methods section for more details) (Hemingway et al., 2005). A total of 3341 participants had complete data on HRV at phase 5 and participated in cognitive tests in at least 1 of the 3 waves over the 10 years of follow-up. Excluding participants with a history of diagnosed CHD (*n* = 483) or stroke (*n* = 156) up to phase 5 left a sample of 2702 individuals (1924 men). Of those, 2014 contributed cognitive test data in all 3 phases, 410 contributed data in 2 phases and 278 contributed data in 1 phase.

Table 1 shows baseline descriptive characteristics of study participants in the lowest sex- and age-specific RMSSD and HF-HRV quintile compared with participants in the upper four quintiles. Being in the lowest versus upper quintiles of RMSSD and HF-HRV was associated with older age and more adverse risk factor profiles. Table 1 further presents that at baseline participants in the lowest HRV quintiles had significantly lower scores on phonemic fluency and a higher prevalence of MCI. Those in the lowest versus upper quintiles of RMSSD had significantly lower global cognitive function scores.

### Main analysis

At baseline (differences in the intercept), cognitive performance decreased with age; 1-year greater age was associated with 0.02 SD (−0.03 to −0.02; *p* ≤ 0.001) lower global cognitive score*.* Over the 10 year period, a decline of 0.13 SD (−0.18 to −0.08; *p* ≤ 0.001) was estimated for the global cognitive score. The statistically significant interaction term between age at baseline and time-in-study showed that there was faster cognitive decline among older participants; specifically, 1-year older age was associated with 0.01 SD (−0.02 to −0.01; *p* ≤ 0.001) faster decline in global cognition over the 10 year period. All models were adjusted for sociodemographic characteristics.

#### Global cognitive score

**Fig. 2:**
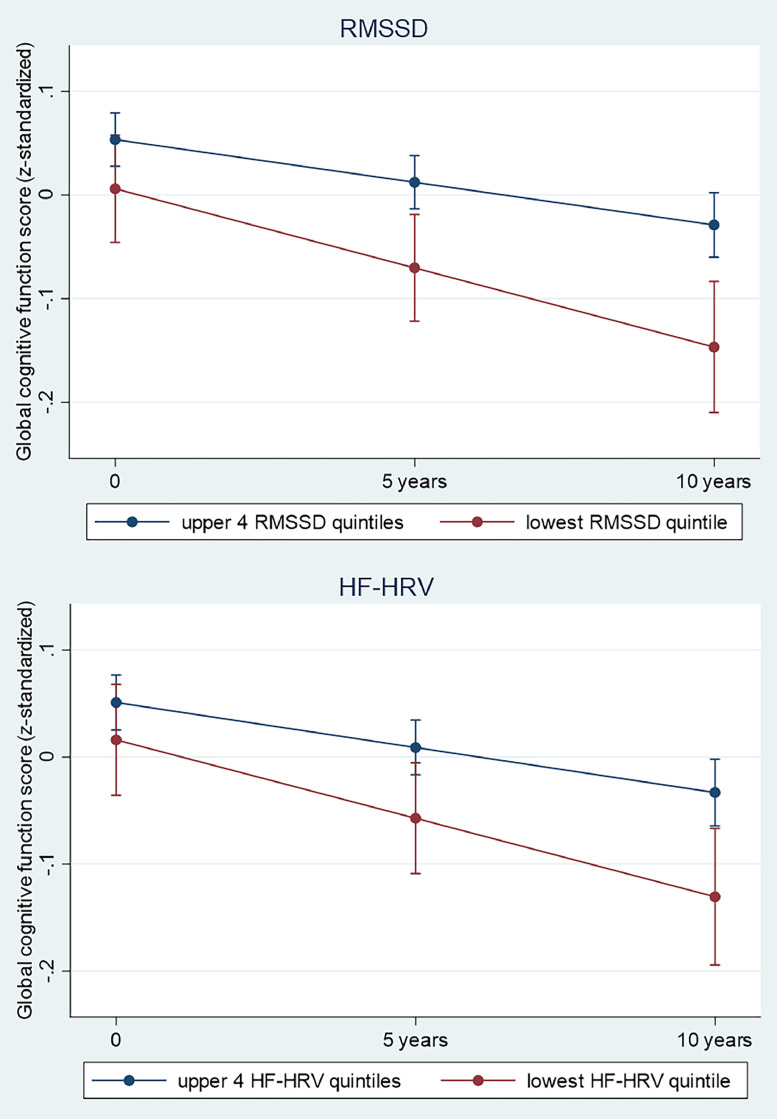
Predicted cognitive change and 95% confidence intervals in adults aged 44 to 69 years by RMSSD (A) and HF-HRV (B) category over a 10 year follow-up period. The estimates of decline for each HRV category were predictions from a linear mixed-effects model including category of HRV (lowest HF or RMSSD quintile at baseline versus upper 4 quintiles), time-in-study, age-at-baseline, sex, ethnicity, education and their statistical interaction with time-in-study.

Table 2 and Fig. 2 present results from the linear mixed-effects models showing associations between baseline HRV/heart rate (1997–1999) and the global cognitive score over the 10 year follow-up period after adjustments for age, sex, ethnicity and education level and their interaction with time (Model 1). In this adjusted model, at baseline (differences in intercept), the associations between HRV and global cognition were not statistically significant.

**Table 2:** Associations (Mixed Models Analysis) between Heart rate variability and Heart rate at baseline (1997–1999) and global cognitive decline over 10 years (1997–1999, 2002–2004, 2007–2009)¹ (*n* = 2702).

			Cross-sectional associations (standardized cognitive score at baseline		Longitudinal associations (change in standardized cognitive score over 10 years)	
		N	β (95% CI)	p	β (95% CI)	p
**Heart rate (bpm)**	**Highest quintile**	541	−0.05 (−0.10 to 0.02)	0.139	−0.06 (−0.12 to −0.001)	0.048
	**Rest**	2161	0.00 (Ref)		0.00 (Ref)	
**RMSSD (ms)**	**Lowest quintile**	541	−0.05 (−0.11 to 0.01)	0.108	−0.07 (−0.13 to −0.01)	0.018
	**Rest**	2161	0.00 (Ref)		0.00 (Ref)	
**HF-HRV (ms^2^)**	**Lowest quintile**	541	−0.04 (−0.09 to 0.02)	0.238	−0.06 (−0.12 to −0.004)	0.037
	**Rest**	2161	0.00 (Ref)		0.00 (Ref)	

¹In those without diagnosed coronary heart disease and stroke up to baseline (phase 5: 1997–1999). Estimates for cross-sectional and longitudinal associations were obtained from linear mixed models including time-in-study, age, sex, ethnicity and highest educational level and their interaction with time-in-study. HF-HRV indicates high-frequency HRV; RMSSD indicates root mean square of successive differences of normal-to-normal RR intervals.

In longitudinal analyses (differences in rate of 10-year change), Table 2 and Fig. 2 further show accelerated decline in global cognitive function in participants in the lowest quintiles of HRV measures and in the highest quintile of heart rate. Specifically, for the main HRV variables, global cognitive function declined by 0.07 SD (−0.13 to −0.01; *p* = 0.018) and 0.06 SD (−0.12 to −0.004; *p* = 0.037) faster for participants in the lowest versus upper quintiles of RMSSD and HF-HRV, respectively. Furthermore, global cognitive function declined by 0.06 SD (−0.12 to −0.001; *p* = 0.048) faster for participants in the highest versus lower quintiles of heart rate. As 1-year older age was related to 0.02 SD lower global cognition (adjusted for sociodemographics), the differences in the estimated 10-year decline in global cognition for participants in the lowest versus upper quintiles of RMSSD and HF-HRV are equivalent to a difference in chronological age of 3.5 years (95% CI: 4.3 to 0.5 years) and 3 years (95% CI: 4 to 0.2 years), respectively.

Table 3 shows that further adjustments for heart rate and its interaction with time (Model 2) did not lead to any appreciable change in the estimated differences in the rate of cognitive decline in participants in the lowest versus upper quintiles of RMSSD and HF-HRV. Likewise, results did not change after adjustment for known confounders including lifestyle factors (physical activity, cigarette smoking status and alcohol consumption: Model 3), cardiometabolic conditions (hypertension, obesity, diagnosed diabetes, and depressive symptoms: Model 4), prescribed medication use (Model 5) or after adjustment for all confounders (Model 6).

**Table 3:** Explanatory models of longitudinal associations (Mixed-Models Analyses) between log-transformed HF-HRV and RMSSD (1997–1999) and cognitive decline over 10 years (1997–1999, 2002–2004, 2007–2009) (*n* = 2702).

		β (95% CI)											
		Model 1	p	Model 2	p	Model 3	p	Model 4	p	Model 5	p	Model 6	p
**Heart rate (bpm)**	**Highest quintile**	−0.06 (−0.12 to −0.001)	0.048			−0.06 (−0.12 to −0.005)	0.033	−0.06 (−0.12 to −0.001)	0.056	−0.06 (−0.12 to 0.001)	0.052	−0.06 (−0.12 to −0.002)	0.041
	**Rest**	0.00 (Ref)		0.00 (Ref)		0.00 (Ref)		0.00 (Ref)		0.00 (Ref)		0.00 (Ref)	
**RMSSD (ms)**	**Lowest quintile**	−0.07 (−0.13 to −0.01)	0.018	−0.08 (−0.14 to −0.01)	0.028	−0.07 (−0.13 to −0.01)	0.022	−0.07 (−0.13 to −0.01)	0.021	−0.07 (−0.13 to −0.01)	0.021	−0.07 (−0.12 to −0.01)	0.030
	**Rest**	0.00 (Ref)		0.00 (Ref)		0.00 (Ref)		0.00 (Ref)		0.00 (Ref)		0.00 (Ref)	
**HF-HRV(ms^2^)**	**Lowest quintile**	−0.06 (−0.12 to −0.004)	0.037	−0.06 (−0.13 to −0.002)	0.058	−0.06 (−0.12 to −0.01)	0.049	−0.06 (−0.12 to −0.02)	0.043	−0.06(−0.12 to −0.001)	0.045	−0.06 (−0.12 to −0.004)	0.066
	**Rest**	0.00 (Ref)		0.00 (Ref)		0.00 (Ref)		0.00 (Ref)		0.00 (Ref)		0.00 (Ref)	

Estimates were obtained from linear mixed-effects models including time, agegroup at baseline, time × agegroup, HF-HRV or RMSSD quintile, and time × HF-HRV or RMSSD group. Table 3 shows the coefficients for the time × HF-HRV or RMSSD group interaction term. The interaction term for models including HF-HRV shows the estimated difference in the 10-year rate of change between those with HF-HRV in the lowest versus upper quintiles. Likewise, the interaction term for models including RMSSD shows the estimated difference in the 10-year rate of change between participants with RMSSD in the lowest versus upper quintiles. HF-HRV indicates high-frequency heart rate variability; RMSSD indicates root mean square of successive differences of normal-to-normal RR intervals. The cut-offs for the highest and lowest Heart rate / HRV quintiles are shown in the notes to Table 1.

Model 1: analysis adjusted for age, age × time, sex, ethnicity and education and their interaction with time.

Model 2: Model 1 + heart rate, heart rate × time.

Model 3: Model 1 + physical inactivity, high alcohol intake, current smoker and their interaction with time (physical inactivity × time, high alcohol intake × time, current smoker × time).

Model 4: Model 1 + cardiometabolic risk factors (diabetes, hypertension, obesity and depressive symptoms), cardiometabolic risk factors × time.

Model 5: Model 1 + prescribed medication use, prescribed medication × time.

Model 6: Model 1 + all covariates from Model 3 – 5 (health behaviours, cardiometabolic risk factors, prescribed medication use), all covariates × time.

Analyses using baseline HRV measures and heart rate as continuous rather than binary variables showed no statistically significant association with the global cognitive scores (*p* > 0.238).

#### Domain-specific cognitive scores

When testing cross-sectional associations of HRV with the five specific cognitive function domains, at baseline (differences in intercept) lower scores on phonemic fluency were observed in the lowest versus upper quintiles of RMSSD (β = −0.10, 95% CI: −0.19 to −0.01; *p* = 0.024). Additionally, being in the highest versus lower quintiles of heart rate was associated with lower phonemic fluency (β = −0.09, 95% CI: −0.18 to −0.002; *p* = 0.044) (N Table 1).

In longitudinal analyses (differences in the estimated 10-year rate of change), similar β coefficients as for the global cognitive score were observed for a number of the domain-specific scores. Longitudinal associations with the two main HRV measures and heart rate were statistically significant only for the Mill Hill vocabulary test (HRV: *p ≤* 0.006; heart rate: *p =* 0.018). For example, participants in the lowest versus upper quintiles had 0.10 SD (95% CI: −0.17 to −0.03; *p* = 0.006)) and 0.12 SD (95% CI: −0.19 to −0.04; *p* = 0.002) faster decline in vocabulary for RMSSD and HF-HRV, respectively.

### Additional analysis

Table 4 presents results pertinent to the secondary aim of our study which used low cognitive function at follow-up as the outcome (being in the lowest sex- and age-specific quintile). Compared with those in the upper 4 quintiles, participants with RMSSD in the lowest quintile had 1.37 higher odds of low cognitive function at follow-up (OR = 1.37, 95% CI: 1.03 to 1.80), after adjustment for age, sex, ethnicity and education. Likewise, those in the highest quintile of heart rate had 1.42 times higher odds of low cognitive function at follow-up (OR = 1.42, 95% CI: 1.07 to 1.88). HF-HRV was marginally associated with higher odds of low cognitive function at follow up (*p* = 0.097).

**Table 4:** Association between HRV and heart rate at baseline (1997–1999) and odds of low cognitive function (lowest quintile) at follow-up (2007–2009) (*n* = 2189).

		N	Model 1 OR (95% CI)	p	Model 2 OR (95% CI)	p
**Heart rate (bpm)**	**Highest quintile**	439 1750	1.42 (1.07 to 1.88)	0.016	1.44 (1.08 to 1.93)	0.013
	**Rest**		1.0 (Ref)		1.00 (Ref)	
**RMSSD (ms)**	**Lowest quintile**	439 1750	1.37 (1.03 to 1.80)	0.029	1.40 (1.05 to 1.86)	0.021
	**Rest**		1.0 (Ref)		1.00 (Ref)	
**HF-HRV (ms^2^)**	**Lowest quintile**	439 1750	1.27 (0.96 to 1.69)	0.097	1.30 (0.97 to 1.74)	0.076
	**Rest**		1.0 (Ref)		1.00 (Ref)	

OR: odds ratios.

Model 1: ORs adjusted for age, sex, ethnicity and educational level at phase 5.

Model 2: ORs adjusted for age, sex, ethnicity and educational level, health behaviours (smoking and alcohol habits, physical activity), cardiometabolic conditions (obesity, depressive symptoms), medication use.

### Sensitivity analysis

The results of our primary analyses were not appreciably altered in the sensitivity analyses which first excluded *n* = 12 participants with mild cognitive impairment (MMSE score<24) at phase 9 (a small number due to the limited number of individuals with baseline MMSE assessement) and *n* = 923 participants with diagnosed diabetes, hypertension, obesity or depressive symptoms at baseline (i.e., restricting our analysis to a subset of 1779 healthy participants). The results revealed similar longitudinal associations of low HRV on faster cognitive decline (e.g. RMSSD: β=−0.08; 95% CI: −0.14 to −0.01; *p* = 0.036). Further sensitivity analyses assessing different percentages (25% or 33% - ie, lowest quartile or tertile) of participants being classified as having low HRV produced findings broadly similar as the main analyses. Compared with the rest, being in the lowest quartiles/tertiles of RMSSD was associated with faster global cognitive decline (*p ≤ 0.047).* For HF-HRV, the corresponding associations did not persist or only reached a marginal level of statistical significance (*p* = 0.068).

## Discussion

Based on a large UK longitudinal study on individuals aged 44 to 69 years at baseline, without prevalent diagnosed CHD or stroke, our investigation suggests that low HRV was empirically associated with faster cognitive decline and lower subsequent cognition over a 10-year period. The following features characterise our findings. First, both vagally-mediated HRV measures, RMSSD and HF-HRV, were associated with a faster decline in global cognitive function. Additionally, high heart rate was linked to faster global cognitive decline. Over a decade, the longitudinal associations observed herein correspond to an estimated age effect of approximately 3 years. However, causality was not demonstrated in our analysis. Second, these associations were independent of baseline sociodemographic and lifestyle factors, cardiometabolic conditions, medication use and faster cognitive decline in older age. Third, when assessing the cognitive domains separately, a decline in vocabulary was most clearly associated with low HRV, while decline in reasoning capacity was marginally linked to low RMSSD. Fourth, low midlife RMSSD was significantly associated with higher odds of low cognitive performance at follow-up. Lastly, of the two vagally-mediated HRV measures we examined, RMSSD exhibited a more robust and consistent association with cognition compared to HF-HRV, as shown through a series of additional and sensitivity analyses.

Although several previous longitudinal studies have demonstrated an inverse association between HRV and cognition (Mahinrad et al., 2015; Schaich et al., 2020; Zeki Al Hazzouri, Elfassy, Carnethon, Lloyd-Jones & Yaffe, 2018), one study did not find such an association (Britton et al., 2008). To our knowledge, no prior research has examined the association of low HRV and subsequent rate of cognitive change in large samples of middle-aged populations. One previous analysis (Mahinrad et al., 2016) based on participants aged 75 years in the Prospective Study of Pravastatin in the Elderly at Risk, reported that over a period of 3.8 years, 10 second SDNN, an index of global autonomic function, was associated with a steeper decline in processing speed. However, the follow-up period was short and the sample relatively old, so preclinical dementia could not be ruled out, and no other measures were evaluated in this study. It is interesting that in another analysis of the Whitehall II study, high IL-6 levels in midlife preceded a greater decline in reasoning over 10 years and were associated with higher odds of decline in global cognition (measured by MMSE) (Singh-Manoux et al., 2014). The vagus nerve is known to play a homeostatic role in relation to the inflammatory response by being involved in anti-inflammatory reflex (Tracey, 2002), and recent meta-analyses have established a strong association between HRV (including RMSSD and HF-HRV) and inflammation (Williams et al., 2019). Therefore, our results broadly complement the findings of this earlier study (Singh-Manoux et al., 2014).

At baseline, global cognitive function showed an association with RMSSD only in the unadjusted model. However, a clear cross-sectional association was observed of RMSSD and heart rate with phonemic fluency, a test primarily relying on executive functions. This aligns with a meta-analysis of 13 studies (Magnon et al., 2022) indicating a small but positive cross-sectional link between vagally-mediated HRV and executive function. Moreover, a meta-analysis of neuroimaging studies demonstrated that higher HRV and inhibitory control of the heart are linked to increased neuronal activity in the prefrontal cortex, a region crucial for executive functions (Thayer, Ahs, Fredrikson, Sollers & Wager, 2012), while lower resting HRV is related to reduced regulation of prefrontal activity (Park & Thayer, 2014). Previous research has demonstrated that tasks involving phonemic fluency and inductive reasoning predominantly engage the prefrontal cortex (Alvarez & Emory, 2006; Goel & Dolan, 2004), while verbal ability and intelligence activate both the prefrontal cortex and the temporal lobe (Ojemann, Schoenfield-McNeill & Corina, 2002). Although we observed differential decreases over time across the individual tests related to executive functions, our findings of accelerated decline in the global cognitive function score and in verbal ability among individuals with low HRV, suggest a likely connection between levels of HRV and executive function in the Whitehall II cohort.

The cutoffs for vagally mediated HRV measures associated with faster cognitive decline were RMSSD values below 10.4–15 ms and HF-HRV values below 37–86 ms^2^, depending on age and sex. RMSSD, less influenced by respiration (Shaffer & Ginsberg, 2017), exhibited a more robust association with cognition in our study. A study (Jarczok, Koenig, Wittling, Fischer & Thayer, 2019) of just under 10,000 adults found that daytime RMSSD values below 15 ms were associated with elevated odds ratios for various cardiovascular risk factors, including inflammatory markers, blood lipids, glucose, and blood pressure. Vagally-mediated HRV could serve as an easily assessable biomarker not only for cardiovascular risk but also for cognitive decline. While disturbed cardiovascular health, primarily in midlife, is a risk factor for cognitive impairment (Ng et al., 2013; Yaffe et al., 2014), our study intentionally focused on middle-aged participants without prior diagnosed CHD and stroke at baseline. We observed that the empirical association between HRV and cognitive decline remained even after accounting for major factors such as age, educational status, sex, and prevalent baseline cardiovascular risk factors like hypertension, obesity, depression, and diabetes as well as other heart disease, lifestyle factors, and medication use. However we ackwnowledge that our results may have been affected by a number of unmeasured factors relevant to autonomic control and cognitive decline, including experiences of traumas during childhood, loneliness, and social isolation. Additionally, adjusting for heart rate did not appreciably alter the HRV-cognitive decline association. Higher heart rate itself was significantly linked to accelerated global cognitive decline and subsequent low cognitive function at follow-up, which aligns with findings from a previous study where a resting heart rate above 80 bpm predicted faster cognitive decline, as measured by MMSE, and an increased dementia risk (Imahori et al., 2022).

There are several potential mechanisms through which autonomic nervous system and vagal regulation may be associated with cognitive function, although the observational nature of our study means that we could not determine causality. Besides the above mentioned anti-inflammatory reflex of vagus nerve, vascular conditions may be involved; sympathetic and parasympathetic activity interact to maintain blood pressure within a normal range and optimise brain blood perfusion (Sloan, Shapiro, Bagiella, Myers & Gorman, 1999). Disturbance of this regulation, leading to increased blood pressure variability, has been associated with cognitive problems (De Heus et al., 2021) and structural brain changes. Neurodegenerative processes in the brain may also contribute to both autonomic dysfunction and cognitive impairment (Femminella et al., 2014): various central nervous system structures affected in dementia are important for autonomic, cognitive and emotional regulation (Critchley et al., 2013); these structures, part of the central autonomic network, encompass the hypothalamus, amygdala, insular and ventromedial prefrontal cortex, and brainstem. For instance, in the brainstem, the nucleus of the solitary tract (Breit, Kupferberg, Rogler & Hasler, 2018) receives afferent vagal nerve fibers, that further project to the locus coeruleus, the source of norepinephrine (NE) for the brain (Breit et al. 2018,; Slater and Wang, 2021). NE release in various brain regions plays a critical role in many aspects of cognition, including attention, memory, executive function, and mood regulation. Furthermore a recent meta-analysis of 41 neuroimaging studies revealed insular atrophy in major neurodegenerative diseases including Alzheimer´s disease, which in turn contributes to cognitive and neuropsychiatric deficits (Fathy et al., 2020). Lastly, deficit in central cholinergic function, observed in dementia and also essential for autonomic control, may affect both autonomic and cognitive pathways (Ferreira-Vieira, Guimaraes, Silva & Ribeiro, 2016).

Although the exact underlying mechanisms remain largely unknown, the faster decline in global cognition and verbal ability observed in our study may tentatively suggest that insufficient vagus nerve modulation in midlife may precede changes in the chemical, physiological and metabolic profile of brain regions, including the brainstem, midbrain, and/or cortex. These changes could result in atrophy, primarily in the frontal lobes, with decreased NE or acetylcholine concentration, associated with dementia and cognitive impairment (Holland, Robbins & Rowe, 2021). Consequently, vagal pathways may play crucial roles in preventing cognitive ageing and could be intervention targets. For instance, maintaining a healthy lifestyle in middle age, including regular physical activity, not smoking, moderate consumption of alcohol and maintaining a healthy body weight have been associated with higher subsequent vagally-mediated HRV over a 10-year period (Jandackova, Scholes, Britton, Steptoe, 2019) and lower risk of dementia (Livingston et al., 2020). Furthermore improvements in health behaviours (in particular increases in physical activity), breathing at resonance frequency (i.e. 4.5–6.5 breaths/minute), HRV biofeedback, medication use (e.g. betablockers) or vagus nerve stimulation have shown some efficacy in improving vagal modulation and/or cognitive outcomes (Cibulcova, Koenig, Jackowska & Jandackova, 2024; Dedoncker, Vanderhasselt, Ottaviani & Slavich, 2021; Göçmen, Aktop, Pinar, Toktas & Jandackova, 2023; Jackowska, Koenig, Vasendova & Jandackova, 2022; Jandackova,Scholes, Britton, Steptoe, 2019; Laborde et al., 2022; Livingston et al., 2020). Particularly promising are healthy lifestyle practices, deep breathing and HRV biofeedback techniques, and non-invasive vagus nerve stimulation, due to their cost-effectiveness and non-invasive nature.

### Strengths and limitations

A key strength of our investigation is the use of participant-level longitudinal data with three repeated measures of cognitive function over a decade in a large nonclinical population. The battery of cognitive tests used in the Whitehall II study is suitable for capturing variability in cognitive task performance. We assessed HRV using 5-minute ECG recordings, considered the gold standard for short-term HRV measurement, which has been shown to be highly repeatable (Schroeder et al., 2004) and sufficient for reliable assessment of RMSSD and HF-HRV, and vagal modulation of heart rate (Laborde et al., 2017; Shaffer & Ginsberg, 2017; Task Force, 1996). Despite the Whitehall II study being occupation-based and thus healthier on average than the UK general population, previous research (Batty et al., 2014) has shown that etiological findings and associations between risk factors and cardiovascular disease from the Whitehall cohort are comparable to those of a UK-wide general population study (British Regional Heart Study) and an iconic, community-based US study (Framingham). However, the rates of cognitive decline in the Whitehall II cohort may be slower than among the general population, potentially explaining at least in part the marginal association between HRV and decline in reasoning. Women and non-white ethnic groups were underrepresented in our study, placing some limitations on the generalisability of our results to wider populations. Other limitations include attrition, missing data, and potential for residual confounding from unmeasured factors that may be relevant to both autonomic control and cognitive decline. These limitations are common to all observational studies. Lastly, our study is correlational, and so we could not establish any causal relationship between vagal modulation and cognitive task performance at baseline or over time.

## Conclusion

In conclusion, our results show that among middle-aged individuals without prevalent diagnosed coronary heart diseases and stroke, low vagal modulation of parasympathetic activity was associated with faster decline in global cognitive function over the 10-year follow-up. Cognitive decline in those with low HRV was estimated to be 3 years faster per decade compared to their counterparts. A decline in vocabulary was more strongly linked to HRV than other cognitive measures. Vagal withdrawal may play an etiological role in cognitive impairment and neurodegeneration and may be a marker of faster cognitive decline. Improvement in vagal modulation has potentially important future applications in the prevention and treatment of cognitive dysfunction.

## Consent statement

The University College London ethics committee approved the Whitehall II study and all participants gave informed consent. Whitehall II data, protocols and other metadata are available to bona fide researchers for research purposes (details on the data sharing policy are available at https://www.ucl.ac.uk/psychiatry/research/mental-health-older-people/whitehall-ii/data-sharing).

## Funding sources

This study was supported by the 10.13039/501100001824Czech Science Foundation (registration number: GACR17–22346Y ). This article has been produced also with the financial support of the European Union under the „Life & Environment Research Center Ostrava“ (LERCO) project (CZ.10.03.01/00/22_003/0,000,003) via the Operational Programme Just Transition, and by the project „Research of Excellence on Digital Technologies and Wellbeing CZ.02.01.01/00/22_008/0,004,583“ which is co-financed by the European Union.

## Declaration of competing interest

The authors declare that they have no known competing financial interests or personal relationships that could have appeared to influence the work reported in this paper.

## Appendix Supplementary materials

Image, application 1
