# Supplementary material for: Midlife heart rate variability and cognitive decline: A large longitudinal cohort study
Source: Int J Clin Health Psychol. 2024 Nov 20;24(4):100518. doi: 10.1016/j.ijchp.2024.100518 (PMC11617396; doi:10.1016/j.ijchp.2024.100518)
Supplement: Supplementary file 1 [file mmc1.docx]

**Supplementary Table 1 Associations (Mixed Models Analysis) between Heart rate variability and Heart rate at baseline (1997-1999) and decline in specific cognitive domains over 10 years (1997-1999, 2002-2004, 2007-2009)¹ (n=2,702)**

**Cross-sectional associations (standardized cognitive score at baseline)**

|  |  | β (95% CI) | | | | | | | | | |
| --- | --- | --- | --- | --- | --- | --- | --- | --- | --- | --- | --- |
|  |  | Memory | | Reasoning | | Mill Hill vocabulary | p | Words | p | Animals | p |
| **Heart rate (bpm)** | **Highest quintile**  Rest | -0.04 (-0.13 to 0.05)  0.00 (Ref) | 0.362 | -0.02 (-0.10 to 0.06)  0.00 (Ref) | 0.571 | -0.04 (-0.12 to 0.04)  0.00 (Ref) | 0.311 | -0.09 (-0.18 to -0.002)  0.00 (Ref) | 0.044 | -0.03 (-0.11 to 0.06)  0.00 (Ref) | 0.545 |
| **RMSSD (ms)** | **Lowest quintile**  Rest | -0.02 (-0.11 to 0.07)  0.00 (Ref) | 0.649 | -0.01 (-0.09 to 0.06)  0.00 (Ref) | 0.737 | -0.06 (-0.13 to 0.02)  0.00 (Ref) | 0.154 | -0.10 (-0.19 to -0.01)  0.00 (Ref) | 0.024 | -0.04 (-0.13 to 0.04)  0.00 (Ref) | 0.330 |
| **HF-HRV(ms^2^)** | **Lowest quintile**  Rest | -0.03 (-0.12 to 0.06)  0.00 (Ref) | 0.546 | -0.03 (-0.11 to 0.05)  0.00 (Ref) | 0.451 | -0.03 (-0.11 to 0.05)  0.00 (Ref) | 0.442 | -0.06(-0.14 to 0.03)  0.00 (Ref) | 0.209 | -0.03(-0.11 to 0.06)  0.00 (Ref) | 0.536 |

**Longitudinal associations (change in standardized cognitive score over 10 years)**

|  |  | β (95% CI) | | | | | | | | | |
| --- | --- | --- | --- | --- | --- | --- | --- | --- | --- | --- | --- |
|  |  | Memory | | Reasoning | | Mill Hill vocabulary | p | Words | p | Animals | p |
| **Heart rate (bpm)** | **Highest quintile**  Rest | -0.09 (-0.24 to 0.07)  0.00 (Ref) | 0.283 | -0.04 (-0.11 to 0.04)  0.00 (Ref) | 0.363 | -0.09 (-0.16 to -0.02)  0.00 (Ref) | 0.018 | -0.03 (-0.15 to -0.10)  0.00 (Ref) | 0.699 | -0.07 (-0.19 to 0.06)¨  0.00 (Ref) | 0.283 |
| **RMSSD (ms)** | **Lowest quintile**  Rest | -0.11 (-0.27 to 0.05)  0.00 (Ref) | 0.179 | -0.07 (-0.14 to -0.01)  0.00 (Ref) | 0.086 | -0.10 (-0.17 to -0.03)  0.00 (Ref) | 0.006 | -0.06 (-0.19 to -0.07)  0.00 (Ref) | 0.376 | 0.01 (-0.11 to 0.13)  0.00 (Ref) | 0.872 |
| **HF-HRV(ms^2^)** | **Lowest quintile**  Rest | -0.07 (-0.23 to 0.09)  0.00 (Ref) | 0.376 | -0.06 (-0.14 to 0.02)  0.00 (Ref) | 0.149 | -0.12 (-0.19 to -0.04)  0.00 (Ref) | 0.002 | -0.04 (-0.17 to 0.09)  0.00 (Ref) | 0.576 | -0.001(-0.13 to 0.12  0.00 (Ref) | 0.994 |

¹In those without diagnosed coronary heart disease and stroke up to baseline (phase 5: 1997-1999). Estimates for cross-sectional and longitudinal associations were obtained from linear mixed models including time-in-study, age, sex, ethnicity and highest educational level and their interaction with time-in-study. HF-HRV indicates high-frequency HRV; RMSSD indicates root mean square of successive differences of normal-to-normal RR intervals
